# FBS-Derived Exosomes as a Natural Nano-Scale Carrier for Icariin Promote Osteoblast Proliferation

**DOI:** 10.3389/fbioe.2021.615920

**Published:** 2021-02-26

**Authors:** Ming Dong, Saixuan Wu, Huijun Xu, Xinxin Yu, Lina Wang, Hua Bai, Weidong Niu

**Affiliations:** ^1^School of Stomatology, Dalian Medical University, Dalian, China; ^2^Department of Stomatology, Bozhou People’s Hospital, Bozhou, China

**Keywords:** exosomes, ICA, osteoblast, proliferation, FBS

## Abstract

Icariin is a class IV drug of low solubility, permeability, and poor bioavailability. Synthetic nanomaterials have developed rapidly. However, some literatures point out that synthetic nanomaterials such as liposomes, aptamers, metal nanoparticles, and nanogels have high toxicity and are affected by the reticuloendothelial system or mononuclear phagocyte system. It is known that exosomes could be used as an ideal clinical drug delivery vehicle to avoid the above-mentioned problems to a certain extent. Studies have shown that drugs can be loaded into exosomes by passive and active loading. We used Fetal bovine serum (FBS) exosomes to carry Icariin for the first time in this experiment, FBS exosomes-Icariin (FBS EXO-ICA) more effectively promoted the proliferation of osteoblasts and bone regeneration than Icariin alone. FBS EXO-ICA could become a new nano scale drug formulation for treating diseases associated with bone loss.

## Introduction

Bone loss is the main clinical manifestation of rheumatoid arthritis, osteoporosis, and myeloma (Currey, 2003; Oftadeh et al., 2015). The current research hotspot for the treatment of these diseases is the regeneration of bone tissue. Under the stimulation of injury-regenerative medicine, osteoblasts provide a cell source for bone defect repair, secrete bone-related extracellular matrix, and accelerate the bone repair process. Exosomes (EXO) are tiny vesicles secreted by most cells. The diameter of a typical exosome is about 30–150 nm (Huo et al., 2019; Luo et al., 2019; Qayoom et al., 2019). It has a lipid bilayer membrane structure and is oval or cup shaped. Exosomes contain specific targeting receptor at their surface which makes them perfect for targeted delivery. Over the past three decades, exosomes have been developed as natural nano-scale drug carriers with unique biological advantages (Fonseca et al., 2016; Ghayad et al., 2016; Nguyen et al., 2016; Trelis et al., 2016).

The synthesis cost of nanomaterials such as liposomes, aptamers, metal nanoparticles, and nanogels are high, the stability is poor, and there are clinical problems that cannot be ignored, including the cytotoxicity of the material and the rapid clearance by the reticuloendothelial phagocytic capacity (REPC) and the mononuclear phagocyte system (MPS), Zhao et al. (2016) and Suk and Gopinath (2017). The instability of liposomes is not favorable for long-circulating treatment, controlled release or conservation (Li et al., 2017). The limitations of aptamers include the susceptibility to degradation by nucleases, fast renal clearance, low thermal stability, and the limited functional group diversity (Odeh et al., 2019). The disadvantage of metal nanoparticles is expensive and toxicity (Mathur et al., 2018). Nanogels have several disadvantages such as rapid reduction in permeability, disposal problems, and high sensitivity to environmental conditions (Zhao et al., 2018). As substances produced *in vivo*, exosomes are natural nanovesicles that are highly biocompatible, of low immunogenicity and cytotoxicity, and they are even able to cross the blood–brain barrier, which makes them the ideal clinical drug carrier (Patel and Patel, 2017). Epimedium is a traditional Chinese medicine used to treat bone diseases, such as osteoporosis and rheumatoid arthritis. Icariin (ICA) is the main biologically active pharmaceutical ingredient of Epimedium (Cao et al., 2019; Chen et al., 2019; Hu et al., 2019). It is soluble in ethanol and ethyl acetate, and minimally soluble in water. Niculescu discovered that ICA enhances BMP-2 mediated osteoblast development by downregulating connective tissue growth factor (CTGF) (Niculescu et al., 2019). Cao first discovered that ICA could promote bone marrow mesenchymal stem cell (BMSC) proliferation through ERK and p38 MAPK signaling (Cao et al., 2019). This showed that ICA could be used as a potential therapeutic drug in bone regeneration (Wu et al., 2019). However, according to the biopharmaceutical classification system, ICA is a class IV drug of low solubility, permeability, and poor bioavailability. Previously, ICA has been loaded into Polycaprolacton (PCL)/gelatin nanomaterials to enhance its role in promoting osteogenesis (Xu et al., 2019b). However, the disadvantages of synthesized nanomaterials have made the demand for drug carriers more urgent (Zuo et al., 2019).

**FIGURE 1:**
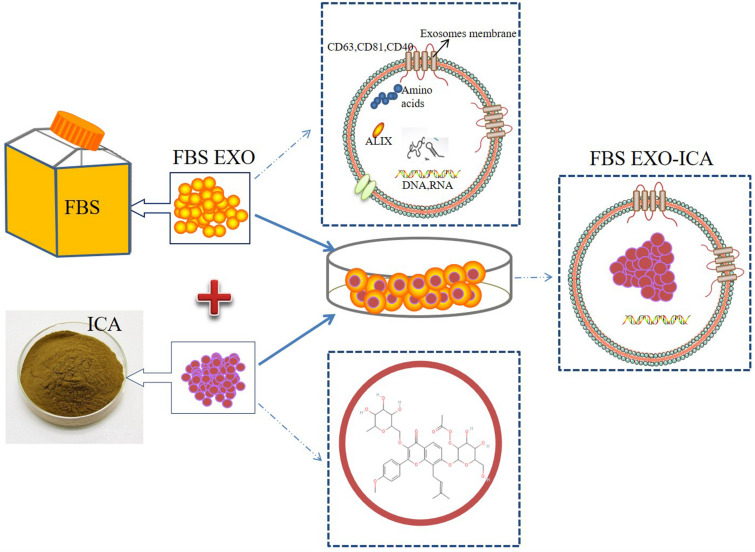
Schematic illustration of construction FBS EXO-ICA.

Exosomes are mainly secreted from immature dendritic cells, mesenchymal stem cells, and other cells (Kalluri and Lebleu, 2020). They have the disadvantages of low concentration, time-consuming preparation, and high cost. Fetal bovine serum (FBS) is an important and commonly used component of cell culture fluids. Studies have shown that FBS contains a large number of extracellular vesicles, and these vesicles have the function of supporting cell growth and reducing cell sensitivity to genetic toxicity and endoplasmic reticulum stress (Ochieng et al., 2009; Eitan et al., 2015; Lehrich et al., 2018). Therefore, in this study an EXO were extracted from FBS and icariin was incorporated into them (FBS EXO-ICA), and the effect of these vesicles on the proliferation of osteoblast precursor cells was observed to provide a new research direction for the clinical treatment of bone loss (Figure 1). This study provides a new approach to the clinical treatment of bone loss by utilizing the effect of icariin loaded into exosomes on the proliferation of osteoblasts.

## Materials and Methods

### Cell Culture

MC3T3-E1 cells (American Type Culture Collection, Manassas, VA, United States) were grown in Dulbecco’s modified Eagle’s medium (DMEM, Invitrogen, Carlsbad, CA, United States) supplemented with 10% FBS, 100 U/mL penicillin, and 100 mg/mL streptomycin. The cells were maintained at 37°C under 5% CO_2_ in humidified air.

### Extraction of FBS Exosomes

Exosomes were extracted from 15 mL of 100% FBS by ultracentrifugation, centrifugation at 2,500 × *g* for 30 min, and centrifugation at 12,000 × *g* for 30 min to remove cell debris, followed by centrifugation at 100,000 × *g* for 3 h to extract the exosomes and other microvesicles. The exosomes were dissolved in 100 μL of PBS solution to prepare a suspension, which was stored at −80°C after measuring the concentration (Beninson and Fleshner, 2015; Eitan et al., 2015; Lehrich et al., 2018).

### Nanoparticle Tracking Analysis

The concentration and particle diameter of the isolated exosomes were measured by Nanoparticle Tracking Analysis (NTA). A 405-nm monochromatic laser beam was applied to the exosome suspension diluted with PBS. Particle motion was analyzed by NTA software. Each particle was identified and tracked frame by frame, and its Brownian motion was tracked and measured. By applying the Stokes-Einstein equation, the particle size was calculated from the speed of particle motion, and the visualized nanoparticle diameter and relative concentration were obtained. All sample evaluations were repeated three times.

### Transmission Electron Microscopy (TEM)

Exosomes (30 μL) were dropped into a petri dish and placed in a copper mesh for 5–10 min. The copper mesh was stained with phosphotungstic acid for 5 min and observed using a transmission electron microscope.

### Western Blotting

Sample were added 100 μl of lysates on ice for 30 min. The cell and exosomes lysates were clarified by centrifugation at 12,000 rpm for 15 min, and the supernatants were collected. The protein concentration was measured with the QuantiPro BCA Assay Kit (KeyGen Biotech Co., Ltd., Shanghai, China). The protein concentration of each sample was measured with the QuantiPro BCA Assay Kit (KeyGen Biotech, Shanghai, China). 20 μg protein was applied to Western Blotting. The membranes were incubated overnight at 4°C with specific anti-CD63 (diluted 1: 200; Abcam, United States), anti-CD81 (diluted 1: 500; Abcam, United States), anti-CD40 (diluted 1: 1000; Bioss, China), anti-ALIX (diluted 1: 1000; Abbexa, United Kingdom), anti-RUNX2 (diluted 1: 500, SAB, United States), anti-BMP-2 (diluted 1: 500, Bioworld, United States), anti-OPN (diluted 1: 1000, Proteintech, United States), and anti-GAPDH (diluted 1: 5000, Bioworld, United States). Incubation with the secondary antibody (diluted 1: 500, ABclonal, China) lasted 1 h. The ECL luminescent solution was configured to collect the blotting results with a BIO-RAD gel imaging system, and the results were analyzed with Image Lab software.

### PKH67 Fluorescence

1 × 10^6^ MC3T3-E1 cells were plated in a six-well plate for culturing and 100 μL of FBS EXO or FBS EXO-ICA were added. 100 μL of the A and B solutions in the PKH67 kit (Sigma, United States) were prepared at a ratio of 1:4000, mix 200 μL, and incubated for 15 min at room temperature in the dark. 200 μL of 1% BSA were added to the above solution, which was centrifuged at 100,000 × *g* for 2 h. The concentration was measured, and the solution was added to the cells at a concentration of 20 μg per well, and cultured for 24 h. The cells were fixed with 4% paraformaldehyde for 20 min, and blocking solution was added for 20 min. Phalloidin (1: 200) was incubated in a wet box at 4°C overnight. Then the cells were stained with DAPI for 8 min prior to observation with an inverted fluorescence microscope.

### Cell Counting Kit-8

Cells were seeded in a 96-well plate at a density of 2,000 per well. After the cells had adhered, 0, 0.1, 1, 10, and 20 μg/mL aliquots of ICA were added to the MC3T3-E1 cells. After incubation for 24 and 48 h, 100 μL Cell Counting Kit-8 (CCK-8) was added to each well. The mixture was incubated for 1 h and the absorbance was measured at 450 nm.

### Preparation of FBS EXO-ICA

Icariin was formulated as a 1 mg/mL stock solution. Icariin solution and exosomes were mixed at a ratio of 1: 9 and incubated for 24 h, then centrifuged at 1,000 × *g* for 10 min to remove free drug. The mixture was centrifuged at 135,000 × *g* for 2 h to collect drug-loaded exosomes (FBS EXO-ICA). They were then dissolved in PBS, filtered through a 0.22-μm filter, and the concentration was measured (Aqil et al., 2016; Munagala et al., 2016; Agrawal et al., 2017).

### High Performance Liquid Chromatography

500 μL of acetonitrile were added to 100 μL of FBS EXO-ICA to destroy the exosomal membrane structure, thereby releasing the drug and precipitating the exosomal proteins. The mixture was centrifuged at 1,000 × *g* for 10 min to isolate the exosomal protein, and the supernatant was used for detection of ICA. The analysis was performed using an Agilent 1200 LC system and an API 3200 LC-triple quadrupole mass spectrometer. A Hypersil ODS C18 column (150 mm × 2.1 mm, 5 μm) was used to analyze a 5-μL sample on the High Performance Liquid Chromatography (HPLC) system. The mobile phase was 0.1% methanol aqueous solution-acetonitrile. ICA was detected by PDA-UV at 200–400 nm, and the drug concentration was calculated with reference to the standard curve of ICA.

### Statistical Analysis

Data are expressed as the Mean ± SEM. Significant differences between test groups were analyzed via one-way analysis of variance and the Student-Newman–Keuls test; *P* < 0.05 was considered to be statistically significant.

## Results

### Extraction and Identification of FBS EXO

**FIGURE 2:**
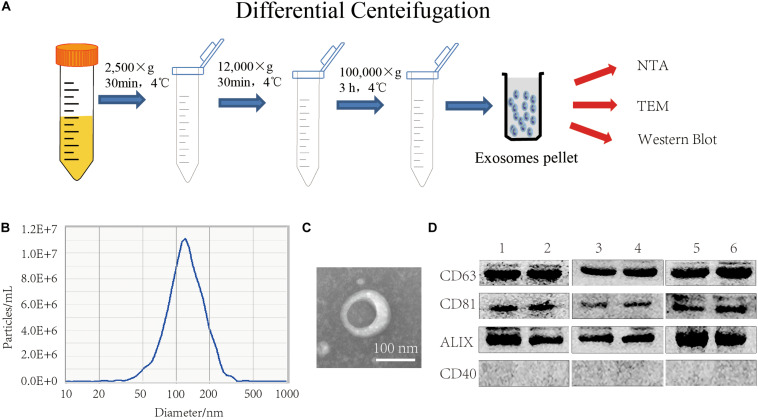
FBS EXO extraction and identification. **(A)** Centrifugation; **(B)** NTA detection of the median particle size of FBS EXO; **(C)** Observation of FBS EXO structure under TEM (×100,000); **(D)** Western Blotting was used to detect the protein expression of exosome markers CD63, CD81, and ALIX and the microcapsule surface marker CD40 in different batches of FBS EXO, 1-6 represented exosomes extracted from different batches.

FBS was centrifuged at 2,500 × *g* and 12,000 × *g* for 30 min to remove dead cells, cell debris, and large vesicles remaining in the serum, and then the exosomes were obtained by centrifuging at 100,000 × g, as shown in Figure 2A. Examination of FBS EXO by NTA showed that the median particle size was 117 nm, which was within the normal range of exosome size (Figure 2B). TEM results showed that FBS EXO had a typical lipid bilayer structure, and the size was between 30 and 150 nm (Figure 2C). This result indicated that the FBS EXO was successfully extracted. Western Blotting results showed that exosome marker factors CD63, CD81, and ALIX were positive; while the microcapsule surface marker CD40 was negative and no bands appeared. This result suggested that FBS EXO was successfully extracted (Figure 2D).

### Effects of ICA on Osteoblast Proliferation

**FIGURE 3:**
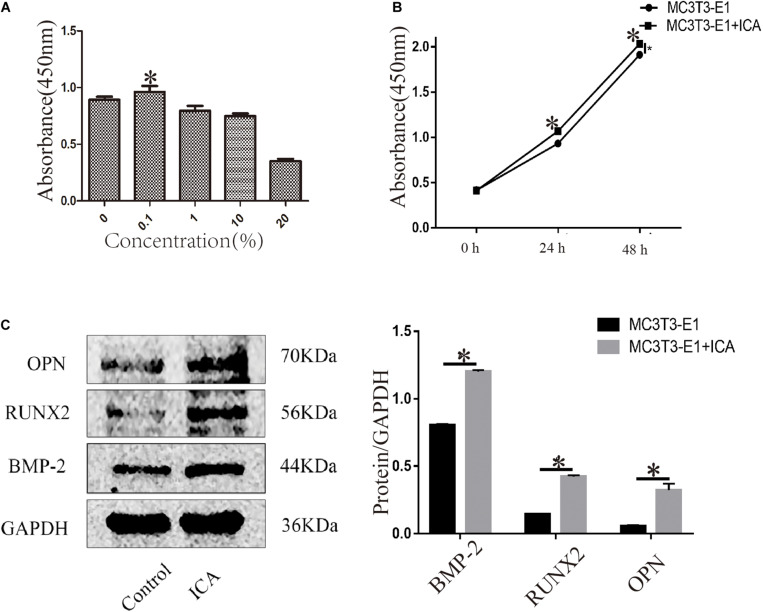
Effects of ICA on osteoblast proliferation. **(A)** ICA (0.1 μg/mL) promoted osteoblast proliferation; **(B)** Time dependence of the effect of ICA on osteoblast proliferation activity; and **(C)** Increased protein expression of BMP-2, RUNX2, and OPN after ICA was applied to MC3T3-E1 cells. *P-value < 0.05 (*n* = 4).

After MC3T3-E1 cells were treated with ICA at 0, 0.1, 1, 10, and 20 μg/mL for 24 h, and CCK-8 results showed that the cell proliferation activity was highest at 0.1 μg/mL, indicating that a low concentration of ICA could promote the proliferation of osteoblasts. As the concentration increased, the cell proliferation activity gradually decreased, and it was the lowest at 20 μg/mL, indicating that a high concentration of ICA was associated with a certain lethality. Thus, 0.1 μg/mL was chosen as the optimal experimental concentration of ICA (Figure 3A). When 0.1 μg/mL ICA was applied to MC3T3-E1 cells for 0, 24, and 48 h, the results showed that ICA treatment of osteoblasts for 24 and 48 h gave a significantly increased cell proliferation activity, and the results were statistically significant (*P* < 0.05) (Figure 3B). Western Blotting showed that the protein expression levels of osteogenic markers BMP-2, RUNX2, and OPN increased significantly after 24 h of treatment with ICA at 0.1 μg/mL. The results were statistically significant (*P* < 0.05) (Figure 3C).

### Construction and Identification of FBS EXO-ICA

**FIGURE 4:**
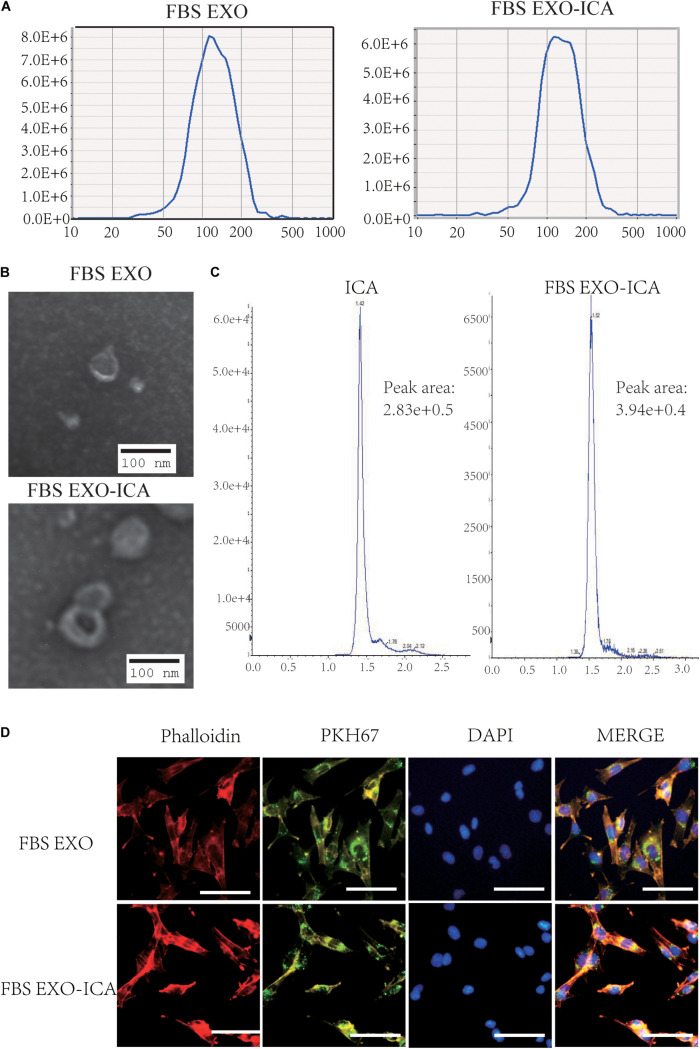
Construction and identification of FBS EXO-ICA. **(A)** Particle sizes of FBS EXO and FBS EXO-ICA detected by NTA; **(B)** TEM results showing the typical lipid bilayer membrane structure and diameter of FBS EXO-ICA (×8,000); **(C)** HPLC indicating the extent of incorporation of ICA in FBS EXO; **(D)** Fluorescence staining showing that phalloidin labeled the cytoskeleton red, PKH67 labeled the exosome membranes green, and DAPI labeled the cell nuclei blue (×200).

The median particle size of FBS EXO detected by NTA was about 117 nm, and that of FBS EXO-ICA was about 122 nm, which is in the normal range of exosomes. The particle size of FBS EXO-ICA was slightly larger than EXO (Figure 4A). TEM results showed that FBS EXO-ICA still had a typical lipid bilayer membrane structure, and at the same magnification, FBS EXO-ICA had a slightly larger diameter than EXO (Figure 4B). The peak area values of the ICA standard solution chromatogram and FBS EXO-ICA were measured by HPLC. According to these values, the efficiency of incorporation of ICA in FBS EXO was about 13% (Figure 4C). PKH67 fluorescence staining was used to detect the uptake of fetal FBS EXO and FBS EXO-ICA by MC3T3-E1. The results showed that both FBS EXO and FBS EXO-ICA were normally taken up by MC3T3-E1 cells (Figure 4D).

### FBS EXO-ICA Promoted the Proliferation of Osteoblasts

**FIGURE 5:**
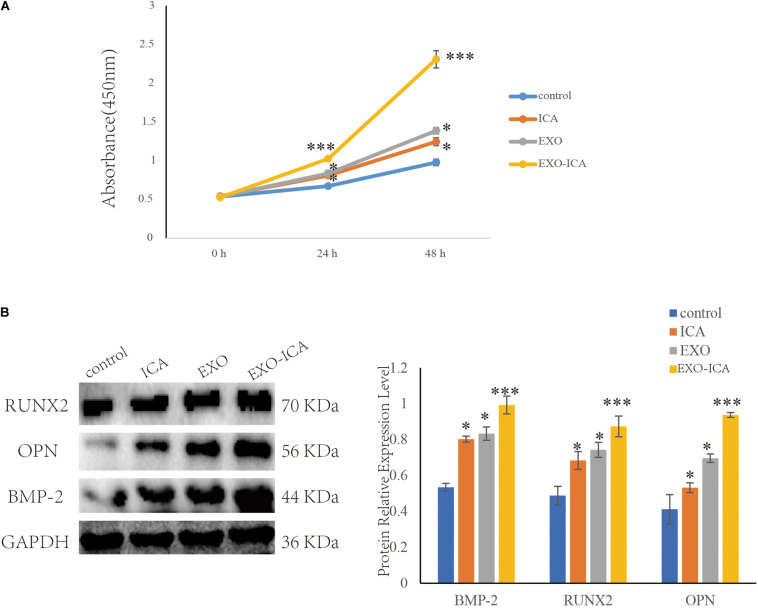
FBS EXO-ICA promoted osteoblast proliferation. **(A)** CCK8 results showed that FBS EXO-ICA had a greater ability to promote the proliferation of osteoblasts than ICA; **(B)** Compared with ICA, FBS EXO-ICA significantly promoted the expression of the osteogenic marker proteins. *P-value < 0.05, ***P-value < 0.001.

Cell Counting Kit-8 results showed the proliferation of cells treated with FBS EXO and ICA were significantly increased compared to those treated with control (*P* < 0.01). FBS EXO-ICA was significantly increased compared with control (*P* < 0.001). The cells treated with FBS EXO-ICA was significantly increased compared to those treated with ICA and FBS EXO (*P* < 0.05) (Figure 5A).

Western Blotting results showed that FBS EXO-ICA, FBS EXO, and ICA increased the protein expression levels of osteogenic markers BMP-2, RUNX2, and OPN more than control (*P* < 0.05). FBS EXO-ICA increased the protein expression levels of osteogenic markers than FBS EXO and ICA (*P* < 0.05) (Figure 5B).

## Discussion

Exosomes are found in fluids such as blood, urine, saliva, amniotic fluid, milk, cerebrospinal fluid, and tears (Wei et al., 2019). EXOs promote bone repair ability, and EXOs were used as carriers to load drugs to promote bone repair. Recent studies have shown that the administration of EXOs can promote endogenous angiogenesis (Zhang et al., 2015), myogenesis (Nakamura et al., 2015) and osteogenesis (Zhang et al., 2019). EXOs have been reported to be effective in the bone regeneration of fractures (Zhang et al., 2020). Narayanan found that the exosomes secreted by HMSC could induce the ossification of HMSC cells *in vitro* and *in vivo* (Narayanan et al., 2016). Cui found that the exosomes of osteoblasts could increase the expression of osteogenic-related miRNAs and promote the differentiation of bone marrow stromal cells into osteoblasts (Cui et al., 2016).

Fetal bovine serum is a supplement commonly used in the laboratory to promote cell growth and cell nutrition. FBS has a high protein content and EXO content (Shelke et al., 2014; Beninson and Fleshner, 2015; Aswad et al., 2016; Lehrich et al., 2018). In this study, NTA, TEM, and Western Blotting were used to determine whether EXO was successfully extracted. Presence of exosomal markers such as CD36, CD63, and XDH in exosomes isolated from milk (Munagala et al., 2016). Studies had also shown that EXO is a natural nanoscale carrier that can participate in cell-to-cell communication and other biological processes. However, some studies have pointed out that the toxicity of synthetic nanomaterials. EXO could be an ideal clinical drug delivery vehicle, as it avoids the above problems. Yu pointed out FBS EXO might serve as efficient carrier systems of immune stimulators to lymph nodes for desired immune responses (Yu et al., 2018).

ICA is the main medicinal ingredient of the Chinese herbal medicine Epimedium, which is a flavonoid (Jiang et al., 2018; Cao et al., 2019; Jing et al., 2019). Epimedium is often used in traditional Chinese medicine to nourish the kidney and strengthen the bones (Kim et al., 2018). Many studies have shown that ICA could promote the proliferation and differentiation of osteoblasts, and regulate the differentiation of mesenchymal cells into osteoblasts (Li et al., 2018; Zhang et al., 2018; Zhu et al., 2018; Xu et al., 2019a). In our experiments, drug carrying was achieved by co-incubating ICA with exosomes, and the successful incorporation of ICA was detected by NTA and TEM. NTA results showed that the particle size of FBS EXO-ICA was slightly larger than that of pure FBS EXO. Farrukh and others embedded triptolide in exosomes and similarly found that the exosomes were slightly larger after drug incorporation. This is consistent with the results obtained in this experiment. TEM results showed the lipid bilayer membrane structure of FBS EXO-ICA, and the particle diameter was slightly larger than that of pure FBS EXO. The above results indicated that FBS EXO was successfully co-incubated with ICA to form FBS EXO-ICA. The efficiency of ICA incorporation in FBS EXO calculated for this experiment was about 13%. Munagala and others loaded various compounds such as Withaferin A (WFA), Paclitaxel (PAC), Docetaxel, PAC (DOC) into exosomes, and the amounts of drug embedded according to UPLC were 5–15% (Munagala et al., 2016). In this experiment, FBS EXO and FBS EXO-ICA were fluorescently labeled with PKH67. The results showed that FBS EXO and FBS EXO-ICA could both be taken up by MC3T3-E1 cells. Ochieng found that FBS EXO could be taken up by tumor cells and recycled back to the conditioned medium (Ochieng et al., 2009). In order to observe whether the ability of ICA to promote osteoblast proliferation was enhanced when it was in exosomes, we compared the efficacies of an ICA group and an FBS EXO-ICA group. CCK-8 results showed the proliferation of cells treated with FBS EXO and ICA were significantly increased compared to those treated with control. This means that both FBS EXO and ICA could promote the proliferation of osteoblasts. The cells treated with FBS EXO-ICA was significantly increased compared to those treated with ICA and FBS EXO. This means that FBS EXO-ICA better promoted the proliferation of osteoblasts than ICA alone. Farrukh demonstrated the chemotherapeutic potential of Celastrol in lung cancer and that exosomal formulation enhanced its efficacy and reduced dose related toxicity (Aqil et al., 2016). Milk-derived exosomes have been investigated for oral delivery of the chemotherapeutic drug paclitaxel (PAC) as an alternative to conventional therapy for improved efficacy and reduced toxicity (Agrawal et al., 2017). The above results indicated that the exosomes loaded with the complex could promote the efficacy of the drug. Bone morphogenetic protein-2 (BMP-2) is an inducer of osteoblast differentiation and bone formation; Runt-related transcription factor 2 (RUNX2) is a specific transcription factor for osteoblast differentiation; Osteopontin (OPN) is involved in bone tissue formation. Therefore, BMP-2, RUNX2 and OPN could be used as the marker factors of osteoblasts. In our results the protein expression levels of the bone markers, BMP-2, RUNX2, and OPN were detected by Western Blotting. The results showed that the protein expression levels of each factor in the FBS EXO-ICA group were significantly higher than those in the ICA and FBS EXO groups. Our results showed that FBS EXO-ICA promoted the proliferation and differentiation of osteoblasts, so the expression of osteogenic marker proteins increased. The above results showed that, compared with ICA, FBS EXO-ICA had a stronger ability to promote osteoblast proliferation and enhance bone regeneration.

In the past two decades, the anti-osteoporosis activity of ICA had become a hot spot in the field of osteoporosis treatment. ICA is classified as a Class IV drug with low solubility, permeability and poor bioavailability in the biopharmaceutical classification system. These unfavorable physical and chemical and pharmacokinetic factors limit its clinical application. Many scholars have attempted to load ICA with nanomaterials to improve its effectiveness, but most research has focused on using synthetic nanoparticles to carry ICA, while endogenous carrier-loaded ICA suitable for oral administration is still quite limited. There is a clinical need for an endogenous drug delivery system carried with ICA to improve its drug efficacy. We successfully extracted FBS EXO and firstly prepared FBS EXO loaded with ICA. Our research showed that the FBS EXO could be used as a carrier to provide small molecules of hydrophilic and lipophilic nature. Due to the low toxicity of FBS, it could potentially enhance the bioavailability of oral drugs and improve the efficacy and safety of the drugs.

## Conclusion

Fetal bovine serum exosomes, a natural nano-scale carrier, could incorporate ICA by co-incubation. FBS EXO-ICA more effectively promoted the proliferation of osteoblasts and bone regeneration than ICA alone. FBS EXO-ICA could become a new nano scale drug formulation for treating diseases associated with bone loss.

## Data Availability Statement

The raw data supporting the conclusions of this article will be made available by the authors, without undue reservation.

## Author Contributions

MD and SW contributed to collection and assembly of the data, data analysis and interpretation, and manuscript writing. HX and XY contributed to computational analysis. LW contributed to provision of study materials, data analysis, and interpretation. HB and WN contributed to conception and design, financial support, manuscript writing, and final approval of the manuscript. All authors contributed to the article and approved the submitted version.

## Conflict of Interest

The authors declare that the research was conducted in the absence of any commercial or financial relationships that could be construed as a potential conflict of interest.
